# Opportunities for Improved Device Design Based on Central Line Placement Practices: Contextual Inquiry Study

**DOI:** 10.2196/84621

**Published:** 2026-02-06

**Authors:** Mary Beth Privitera, Sameer Khan, Bilal Irfan, Shayan Ali, Cecelia Arredondo, Kyrsten Sanderson, Jordan Bonomo

**Affiliations:** 1 University of Cincinnati Know Why Design, LLC Mason, OH United States; 2 Department of Critical Care Medicine Hoag Hospital Newport Beach, CA United States; 3 Department of Epidemiology Department of Neurology University of Michigan Medical School Ann Arbor, MI United States; 4 Center for Surgery and Public Health, Brigham & Women’s Hospital Center for Bioethics Harvard Medical School Boston, MA United States; 5 Neuroscience Department Austin College Sherman, TX United States; 6 North Texas Medical Research Institute Rockwell, TX United States; 7 School of Design, College of Design, Art, Architecture & Planning University of Cincinnati Cincinnati, OH United States; 8 Section Chief, Critical Care Department of Emergency Medicine, Neurology, NeuroCritical Care University of Cincinnati Cincinnati, OH United States

**Keywords:** contextual inquiry, central line placement, medical device design, patient safety, ease of use

## Abstract

**Background:**

Central venous catheters (CVCs) are indispensable to contemporary critical care, perioperative management, and emergency resuscitation, yet their insertion remains fraught with preventable harm and inefficiency.

**Objective:**

This study aimed to identify all areas of CVC placement that can be improved through device design using human-centered design and qualitative research methods.

**Methods:**

This qualitative study was a contextual inquiry of CVC placement, which included observation alongside brief face-to-face interviews with physicians. It was aimed at providing a depth of understanding using evidence to demonstrate causality. This study was conducted at 3 hospitals in the emergency department, the intensive care unit, and the operating rooms. Where possible and with additional consent, sessions were recorded in video or still photography, or at times both. This study included 19 observations and 24 interviews.

**Results:**

In this study, the approach to CVC insertion was consistent across hospitals and care environments, with moderate variability spanning a few sections, such as suture and dressing use or lack thereof in specific care environments. The described and observed difficulties leave room for improvement in device design. The results of this study indicated that there are 34 discrete steps to placing a CVC line, with most time spent during sterile preparation. As a result of the device or kit design, challenges were observed. These included missing essential materials from kits, difficulty distinguishing between nonsterile and sterile items, challenges with lidocaine ampules, patient claustrophobia from draping, and a lack of user preference for kit contents. Additional challenges included obscured ultrasound views, kinked guidewires, overall procedural untidiness, and considerable waste management issues.

**Conclusions:**

An intuitive kit that aligns with predictable human behavior and eliminates unnecessary multistep detours can reduce novice failure rates, cognitive load, and practice inconsistency, and it could also curb nonrecyclable waste from “backup” kits opened for a single missing item. By reframing CVC systems as sociotechnical solutions rather than static assortments of parts, the same design moves that minimize improvisation and coordination errors for physicians may also reduce dwell time and manipulation events for patients, thereby advancing the core triad of safety, procedural efficacy, and everyday usability. By examining how clinicians place central lines, this study reveals modifiable design flaws that perpetuate risk despite decades of procedural standardization. Contextual inquiry provides the evidentiary bridge between clinical imperatives to reduce complications and the practical realities of device use. Embedding such investigations at the outset of design and iteratively throughout product life cycles offers a path toward safer, more efficient, and more humane central venous access for both patients and providers.

## Introduction

Central venous catheters (CVCs) are indispensable to contemporary critical care, perioperative management, and emergency resuscitation, yet their insertion remains fraught with preventable harm and inefficiency. Large contemporary syntheses estimate that approximately 30 in every 1000 patients with a CVC in place for 3 days will experience at least one serious complication, be it arterial cannulation, pneumothorax, central line–associated bloodstream infection (CLABSI), or deep venous thrombosis, whereas catheter malfunction alone occurs at roughly 6 events per 1000 catheter days [1]. Additionally, the insertion process can be a source of complication. CLABSIs still claim thousands of lives each year in the United States and add billions of dollars to health care costs despite decades of prevention bundles and increasingly routine ultrasound guidance intended to curb mechanical mishaps and accelerate successful cannulation [2].

While epidemiological surveillance and randomized trials may have helped refine insertion checklists and sterility protocols, far less attention has been paid to the concrete interactions among users, tools, and environments that shape everyday practice. Human factors frameworks may be an impetus to study actual work as done rather than work as imagined. The US Food and Drug Administration’s 2016 guidance on applying human factors in medical devices and the Association for the Advancement of Medical Instrumentation TIR51 standard on contextual inquiry may provide the grounding for early, field-based observation and interview techniques to surface latent use errors, cognitive burdens, and design mismatches before devices reach market or are iterated for safety [3,4].

This study applied a structured contextual inquiry across 3 hospitals and multiple care settings to showcase how central line placement unfolds in real-world settings minute by minute, from presterile preparation to dressing application. By triangulating in situ observation with brief clinician interviews, 34 discrete steps were catalogued, mapping procedure time distributions and documenting recurrent friction points that compromise sterility, ergonomics, and situational awareness. This study aimed to identify all areas of CVC placement that can be improved through device design. From the drapes that obscure patients’ faces and guidewires that become kinked to the missing of essential or preferred components, this study highlights the basis for next-generation kit architecture, accessory design, and room layout that align with real user needs in both chaotic and controlled environments.

## Methods

### Overview

This qualitative study was a contextual inquiry of CVC placement, which included observation alongside brief face-to-face interviews with physicians. It was aimed at providing a depth of understanding using evidence to demonstrate causality. According to Maxwell [5,6], qualitative research is well suited for causal inference as it allows for detailed examination of specific processes and mechanisms in real-world contexts, revealing how and why outcomes occur beyond mere correlations [5,7]. A detailed description of the process, tools, and people involved is provided below. The technique of contextual inquiry is promoted by the Food and Drug Administration human factors guidance (2016) to determine user needs at the start of any design process. The data collected in this study included both observational and interview data, with 3 main areas of focus: the user, the environment, and the tasks as part of the steps and workflow.

This study was conducted at 3 hospitals in the emergency department (ED), intensive care unit (ICU), and operating rooms (ORs). Where possible and with additional consent, sessions were recorded on video or still photography, or at times both. This study included 19 observations and 24 interviews (Table 1). The total number of observations and interviews conducted at each site was determined by the clinical need and patient-clinician consent at the time of the study. In instances in which patient consent was not provided but physicians wanted to participate, the physicians could opt to demonstrate their placement technique using a simulated patient in the care environment.

**Table 1:** Breakdown of observations and interviews by location.

	Cincinnati, Ohio, n (%)	Wake Forest, North Carolina, n (%)	Sacramento, California, n (%)
Observations (n=19)	9 (47)	7 (37)	3 (16)
Interviews (n=24)	8 (33)	8 (33)	8 (33)

A total of 24 physicians were involved in this study, including emergency medicine, critical care, and anesthesiology specialists. Observations and interviews were conducted in perioperative rooms, ORs, EDs, and ICUs.

Within each hospital, the same brand of kit was used; however, in one instance at Wake Forest, there was a different type of kit used between the OR and the ICU and ED. The kits consisted of other brands and product identification numbers (Table 2).

**Table 2:** Brand of kits used by location and environment of care.

	Cincinnati, Ohio	Wake Forest, North Carolina	Sacramento, California
Operating room	Edwards Multi-Med CVC^a^ 3K20N18141NL	Arrow central venous access kit ASK-21242-PCMH1	Arrow Blue Plus pressure-injectable multilumen CVC kit ASK-45703-PIO
Intensive care unit	Edwards Multi-Med CVC 3K20N18141NL	Arrow pressure-injectable multilumen CVC kit CDC-45703-XP1A	Arrow Blue Plus pressure-injectable multilumen CVC kit ASK-45703-PIO
Emergency department	Edwards Multi-Med CVC 3K20N18141NL	Arrow pressure-injectable multilumen CVC kit CDC-45703-XP1A	Arrow Blue Plus pressure-injectable multilumen CVC kit ASK-45703-PIO

^a^CVC: central venous catheter.

All interviews followed the core principles of contextual inquiry studies in that they were conducted in the users’ real-world environment, with the research team establishing a master (physician)-apprentice (researcher) relationship. As the procedure unfolded, and at opportune times, the researcher would share interpretations to uncover observations and deeper insights. All the interviews were guided by the tasks in the CVC placement procedure and focused on the usability of each element. Typical observation or interview experiences ranged from 45 minutes to 1.5 hours, as influenced by clinical responsibilities and the participants’ availability.

### Ethical Considerations

The study protocol was appropriately reviewed by the University of Cincinnati institutional review board, which determined that it did not constitute human participant research under federal regulations, as the primary focus was on device improvements and quality of care rather than generating generalizable knowledge about individuals. Despite the nonresearch classification, informed consent was required from all participants. As detailed observations were conducted of clinical procedures that could capture sensitive patient information or professional performance details, robust measures were in place to protect privacy and confidentiality. In addition, efforts were made to ensure a diverse participant pool to avoid biased insights that could perpetuate disparities in device design or procedural improvements.

## Results

In this study, the average overall time to place a CVC varied across care environments (Table 3). In discussions with clinicians, some postulated that procedure times vary proportionally with the provider’s perceived control over the patient and environment (ie, that the more chaotic the environment, the longer the procedure).

**Table 3:** Average central venous catheter (CVC) placement timing in different environments of care.

Environment	CVC placement time
Operating room	16 min, 48 s
Preoperative care	20 min, 15 s
Intensive care unit	27 min, 53 s
Emergency department	35 min, 28 s
Overall, mean (SD)	25 min, 6 s (7 min 12 s)

**Figure 1:**
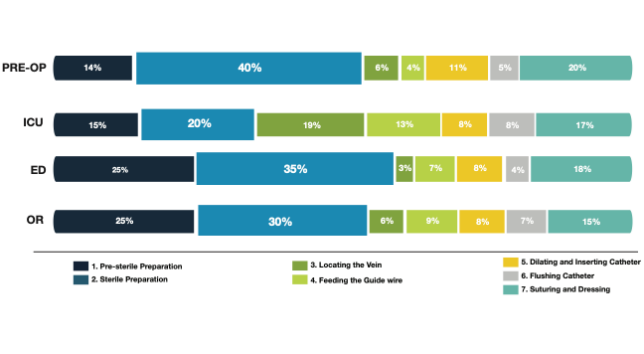
Percentage of time per section of the central venous catheter placement procedure by environment. ED: emergency department; ICU: intensive care unit; OR: operating room.

Procedural timing was also broken down into 7 distinct sections within the overall procedure (Figure 1). These included the following: presterile preparation, sterile preparation, vein localization, guidewire insertion, catheter dilation and insertion, catheter flushing, and suturing and dressing. Of note, time from presterile to sterile preparation accounted for more than half of the overall procedure time in all locations except the ICU. The research team noted that the number of staff members participating in setting up for the procedure varied across sites and care environments.

**Figure 2:**
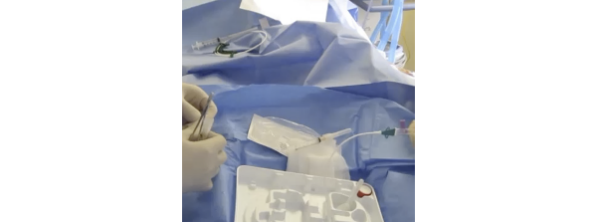
Patient acting as an extended sterile work surface.

The patient experience during CVC placement highlights conditions that impact overall safety, efficacy, and usability. Draping a conscious patient can be problematic. During procedure preparation, the provider unpacks sterile drapes from a kit and prepares a sterile field directly over the patient (Figure 2). This extends the sterile field, albeit an unstable one, directly over the patient. As a result of patient movement, the required equipment for the procedure can fall out of the sterile field or become lost. Uncooperative patients require additional procedures, such as intubation, muscle relaxation, and sedation, before CVC placement.

**Figure 3:**
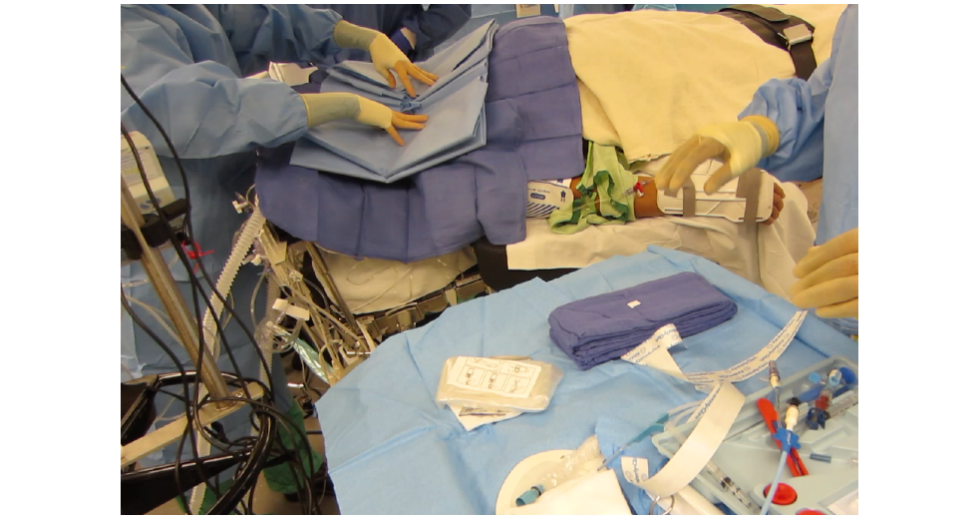
A patient drape placed over the patient’s face in the intensive care unit.

In the OR, the patients were intubated and unconscious. However, in the ED and ICU environments, the patients were often conscious and mobile. In all cases, draping covered the patient’s face throughout the procedure, creating a potentially claustrophobic and challenging environment (Figure 3). In clinical practice, there is substantial variability in body habitus across the patient population. As a result, the drapes provided in the kits may not fit patients classified as obese. In some cases, the aperture provided by the drape is not large enough to accommodate the insertion location, resulting in the provider improvising by creatively engineering a larger opening.

Patient airways require management throughout the procedure. In this study, patients in the ICU were fitted with an oxygen mask. A mask is often placed in case a future procedure is needed to avoid disturbing the sterile field.

**Figure 4:**
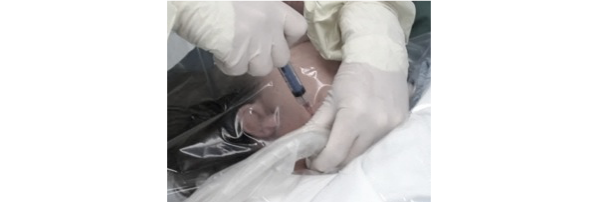
Aperture opening of the drape adhered directly to a patient’s face.

Gaining access and positioning can often lead to patient discomfort. Patients are required to hold awkward Trendelenburg positions with their head turned and extended to optimize their anatomy for successful CVC placement (Figure 4). Patients were instructed to maintain position throughout the procedure, although it can be uncomfortable because the plastic portion of the drape may be directly on a patient’s skin. For conscious patients, this at times required further anesthesia and posed challenges for physicians, given that, as access was gained, patients would move. This position proves challenging for patients who are obese who may also require additional supplies and more extended periods to complete procedure preparation.

**Figure 5:**
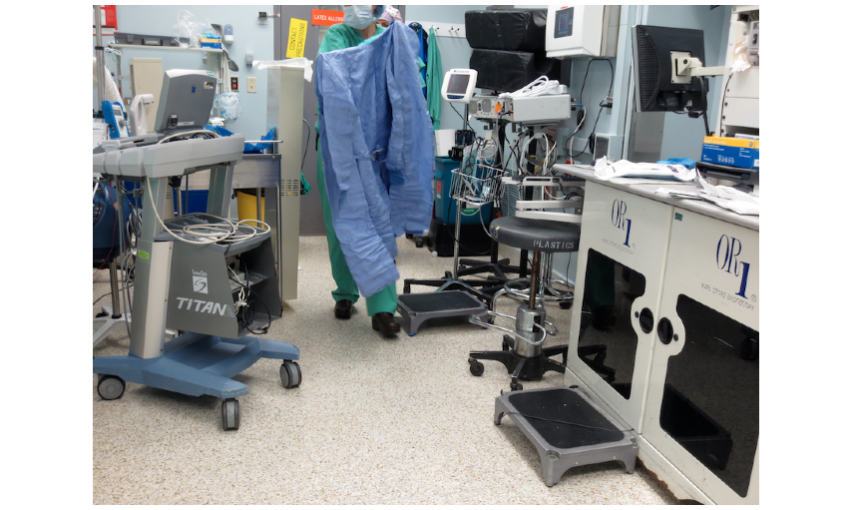
Donning of personal protective equipment in a tight space.

The provider experience during CVC placement also underlines conditions that could impact overall safety, efficacy, and usability. Physicians are required to don personal protective equipment in tight spaces without compromising sterility (Figure 5). In this study, the order of preparation varied across care locations. For example, ED physicians sometimes elected to preclean the patient before gowning, which allowed time for the chlorhexidine preparation to dry. In contrast, in the OR, some physicians prepared personal protective equipment in advance.

In the CVC procedure, 34 individual steps require the provider to locate key pieces of equipment and determine where and how to use them while maintaining a sterile workspace. During this multistep process, physicians often improvise, which may place a heavy cognitive burden on them and require coordination. For example, physicians will think through the procedure before beginning, questioning whether they have all the necessary supplies and where to place them to maximize access while maintaining the sterile field and coordinating with other support personnel.

Across all sites and use environments, at least 2 physicians were present. This enabled 1 sterile operator and 1 support person to adjust patient drapes, including using a towel to protect the patient’s face; retrieve items that were required but not readily available in the kit or forgotten; and sterilely prepare the ultrasound probe.

**Figure 6:**
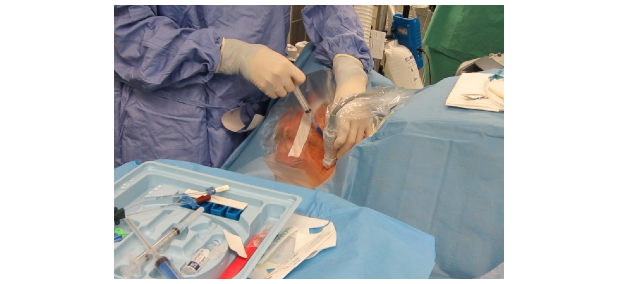
One-handed aspiration while holding the ultrasound probe.

Physicians were often in awkward positions due to suboptimal biomechanics resulting from product design, room layout, or both. For example, physicians struggled to fit all the necessary equipment on a single surface due to space constraints. The equipment is often split between multiple work surfaces (ie, patient and table), forcing the provider to turn to access both surfaces and remove their focus from the patient. The space constraints also make it difficult to avoid touching a nonsterile surface during the procedure. Furthermore, flushing with a standard syringe requires an uncomfortable and awkward hand movement needed to achieve aspiration, as the device is designed to deliver a solution. When ultrasound is used for safe access, both hands are required to hold the equipment, which can make aspiration a challenging one-handed maneuver (Figure 6).

**Figure 7:**
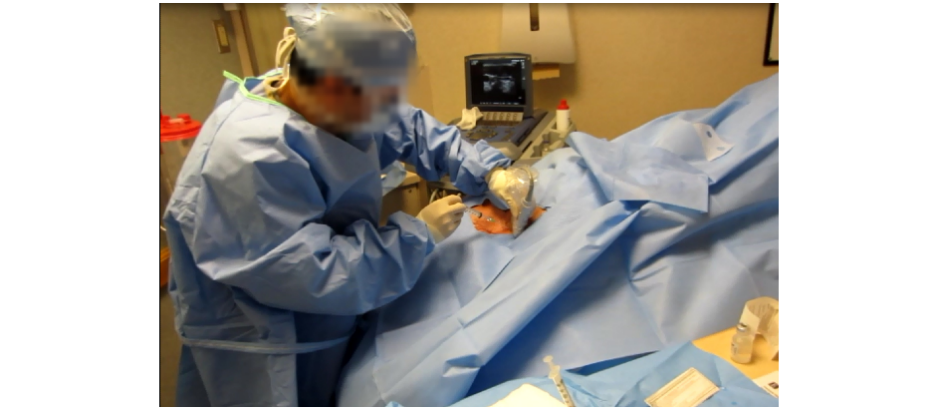
Access needle angle relative to the ultrasound probe.

**Figure 8:**
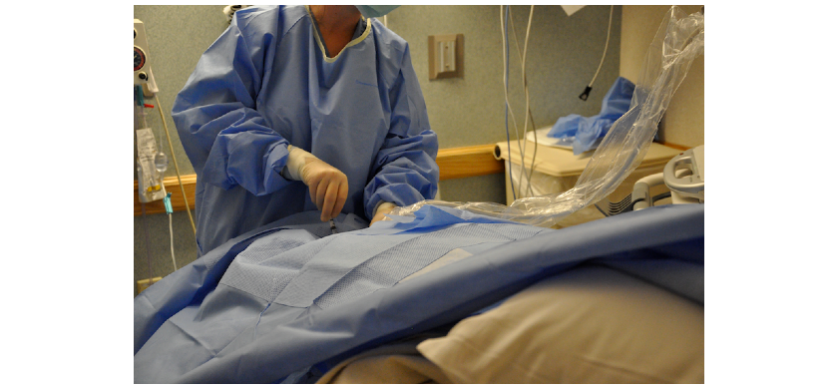
Ultrasound monitor position requires physicians to look away from the access site.

Physicians held the syringe and introducer needle at a 45 to 30° angle and subsequently lowered the angle while advancing the needle (Figure 7). To verify position, physicians would draw more blood and assess color and pressure gradient. Some commented that some step was the highest “stress” point in the procedure. One intensivist even remarked that finding the vein was the most stressful part and that, once the wire was in and the needle was out, their stress subsided. At this point, there is a risk of pneumothorax or perforation of the carotid artery. It was observed that maintaining stability and aspirating to visualize venous blood return is ergonomically challenging for physicians. This is further complicated by the ultrasound monitor being placed outside the provider’s field of view (Figure 8).

Additionally, the pressure that the user applies to the vein with the ultrasound probe can alter central venous anatomy when the device is withdrawn from the skin surface. Physicians were at times compelled to make slow, careful movements, with risk controlled by the user’s experience. Rather than an intuitive device design, advanced troubleshooting or error prevention during the procedure may depend on the provider’s expertise and dexterity.

**Figure 9:**
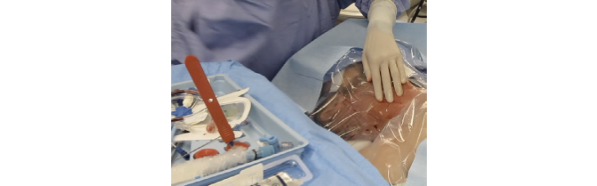
Opening the kit midprocedure with the sharps anchored in the kit.

While simultaneously troubleshooting and preventing errors, physicians are exposed to sharps and biohazards once access to the vein is achieved. For example, users are exposed to the access needle, where the skin incision scalpel is typically contaminated with the patient’s blood within the working area (Figure 9).

**Figure 10:**
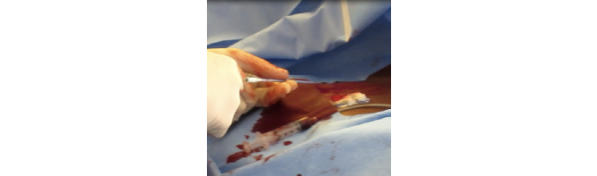
Advancement of the dilator over the wire in slippery conditions due to venous return.

**Figure 11:**
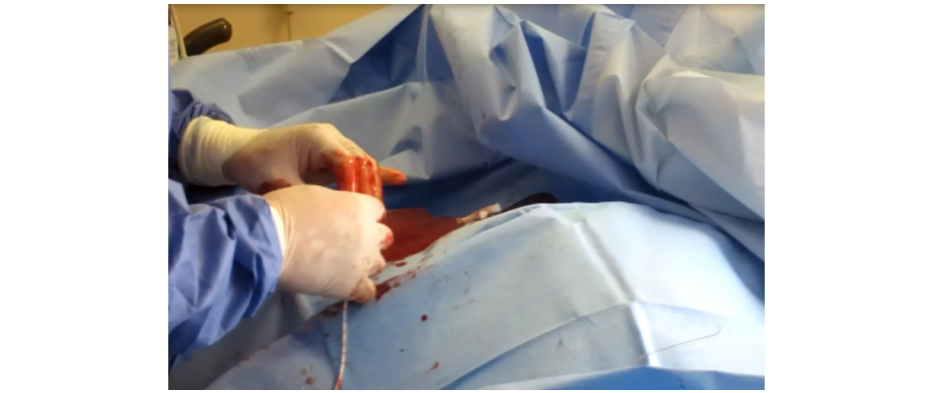
Catheter insertion with exposure to biohazard.

While efforts were made to control and maintain the safety of sharps, the CVC kit design places the cognitive burden, safety, and responsibility on the provider. As vein dilation occurs, the access site often becomes highly disordered and poses a biohazard to health care physicians (Figure 10). Once the vein was dilated, increased blood flow increased the stress and inconvenience of the procedure in that the guidewire itself became a slippery surface. It could become misplaced, requiring additional intervention. This hazardous situation also extends to catheter insertion (Figure 11).

**Figure 12:**
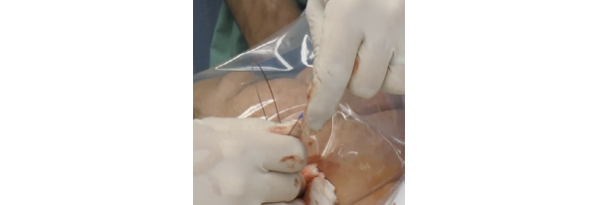
Coiled guidewire enabling control during catheter insertion.

Physicians were required to thread the catheter over the guidewire and adopted a coiling behavior to maintain control and reduce proximal tip movement, which can complicate device alignment (Figure 12).

**Figure 13:**
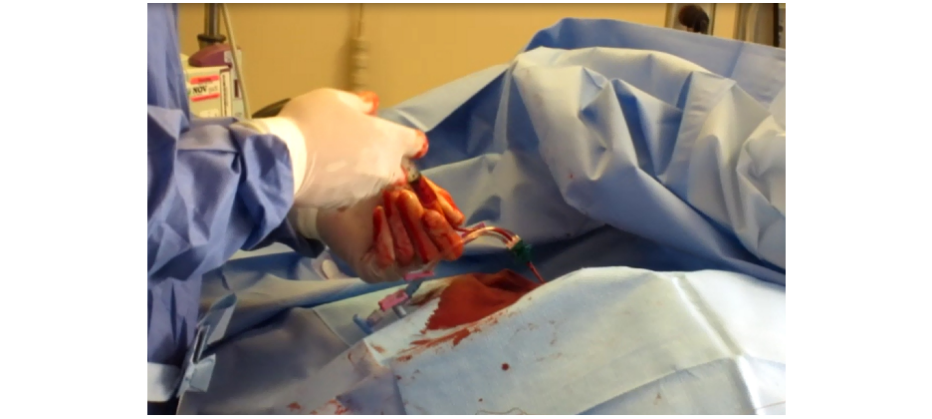
Novice users have increased exposure to biohazards.

These situations were particularly challenging for novice health care providers, who grappled with each step of the procedure (Figure 13). Consequently, inexperienced health care physicians often have unsuccessful attempts to place CVCs.

**Figure 14:**
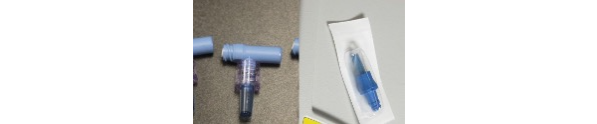
Different forms of valve connectors.

The product design of traditional CVC kits was not always entirely optimized for streamlined assembly. Many kits, for example, did not have all the supplies or the preferred supplies for CVC placement. As a result, clinicians often spent additional time gathering the necessary materials (eg, skin preparation, gauze, saline, and lidocaine) as well as the preferred materials because the valves provided in the kits were at times not compatible with other hospital equipment, thus suggesting better packaging so that the risk or delay in the procedure is minimized. Some users preferred vertical valves such as clave connectors (Figure 14). Physicians who chose to use nonstandard or additional supplies often opened a secondary kit sourced from separate locations within the hospital. Items collected may include additional drapes, Mayo stand, lidocaine, sterile flush syringes, chlorhexidine preparation, and ultrasound devices or supplies.

**Figure 15:**
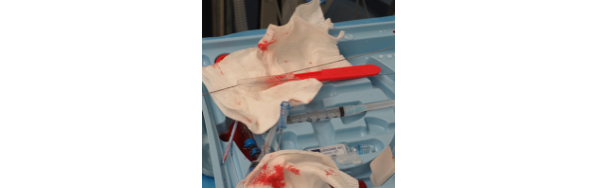
Sharps are placed on top of the kit during the procedure.

When physicians remove the guidewire, there is no proper receptacle to place it in; it is thrown away with nonsharp materials or hazardous materials. In this study, some physicians did not use sharps holders during the procedure, preferring to leave sharps on the tray (Figure 15), suggesting an unmet need among users. For those who used the temporary sharps holder provided in some trays, it was poorly secured and became top-heavy, often leading to dropped sharps. Additionally, there were no safeguards for the disposal of all sharps.

**Figure 16:**
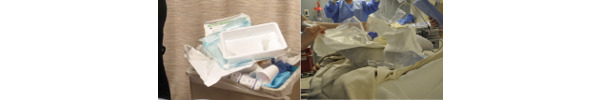
Waste accumulation because of the kit design.

There is a considerable amount of nonrecyclable waste and challenges because of the kit design (Figure 16). In this study, the kits at times lacked a clear delineation between the layers that designate items and specify when they should be used. There were excess materials not used in the procedure, which contributed to the overall waste. Physicians often draped the sterile area and manipulated the drape around the patient’s face and head, making it easier for the patient to breathe and reducing claustrophobia.

**Figure 17:**
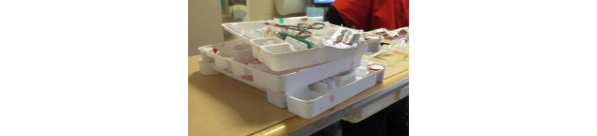
Three kits opened for the guidewire and dilator due to kinking.

The quality and design of the CVC products’ individual elements pose usability challenges, including misalignment with predictable human behavior, heavy reliance on memory, and a lack of visual or tactile references. One challenge in this study was that the guidewire kinked frequently. When this happened, physicians would often try to remove the kink or use the wire regardless. If this guidewire is rendered unusable, the provider may open a new kit to replace the failed one or, in some instances, a missing part (Figure 17).

In this study, the dilator was the same color as the draping and could be camouflaged, eventually getting lost in it, causing health care providers to search. Additionally, due to the complex setup, components sometimes fell outside of the sterile field. Another detail that caused some difficulty in the guidewire design was the “J” curve. While it can provide safe intravascular advancement, this curve on the wire can also make the guidewire more difficult to thread into the catheter. Some physicians were observed making what some may describe as counterintuitive workarounds and flipping the guidewire to the straight end to easily advance it through the catheter. Though well intended, this variation in CVC placement may increase the risk of complications.

Furthermore, there is little to no tactile feedback from the guidewire feeder, making it difficult for the user to know whether the wire is advancing. This is further complicated by blood-stained gloves, which make the guidewire markings difficult to see and grasp. The results of these outdated design decisions can create an unnecessary burden on physicians to judge catheter alignment on the guidewire and insertion depth.

## Discussion

### Principal Findings

In this study, the approach to CVC insertion was consistent across hospitals and care environments, with moderate variability spanning a few sections, such as suture and dressing use or lack thereof in specific care environments. The described and observed difficulties leave room for improvement in device design (Table 4).

**Table 4:** Summary of use-related issues throughout the procedure.

Procedure section and device used			Problem area
**Presterile preparation**			
	Central venous catheter kit	It does not include all essential materials needed for central line placement, resulting in unnecessary delays. It is unable to distinguish nonsterile and sterile items during nonsterile preparation.	
	Nonsterile syringe	Health care providers typically empty 3-5 syringes (30-50 mL) of saline solution into the kit basin from nonsterile saline syringes. These were not included in the kit.	
	Lidocaine	Because the lidocaine is not sterile, health care providers will either have someone hold it for them, tape it to a Mayo cart, or chase down the ampule while loading the syringe.	
**Sterile preparation**			
	Patient drape	Due to the placement of the full-body drape, patient claustrophobia can occur; it is challenging for the provider to maintain sterility and manage the patient during the procedure.	
**Locating the vein**			
	Introducer needle	Physicians expressed a preference for the 18-gauge introducer needle without a catheter over the 20-gauge needle with an 18-gauge catheter assembly. For patients with obesity, physicians noted that the length (2.5 inches) of the needle was not sufficient. The introducer needles were not echogenic; thus, they were difficult to view in the ultrasound monitor.	
	Ultrasound	The ultrasound monitor was not placed within the line of sight of the provider, making it difficult for them to effectively use the tool. Health care providers do not generally document ultrasound use by capturing an image of the procedure as required by major insurers for reimbursement.	
**Feeding the guidewire**			
	Guidewire	Wire kinking while executing the procedure was problematic, and once deemed unusable, the provider would open another kit only to access an additional wire. The “J” tip of the wire was difficult to thread and feed. Physicians had difficulty estimating how much of the wire was inserted. If they were unsure, they took the wire out of the delivery system. Once the wire is removed from the delivery system, it is difficult to reload; thus, once it is out, it typically stays out. Markings on the wire were also not clear to the providers. Due to the inherent messiness of the procedure, blood is often on the providers’ hands, making it difficult to handle the guidewire delivery system.	
**Dilating and inserting the catheter**			
	Dilator	The dilator often kinks if the provider aggressively pushes. If the dilator is no longer usable, another kit is opened only to access another dilator. The dilator length (4 inches) is not sufficient for patients with obesity.	
	Catheter	Threading the catheter over the wire is a difficult task. Physicians have a hand tremor when performing this task. It is difficult for the provider to determine whether the tip of the catheter has reached the superior vena cava. Physicians will estimate placement and follow up by x-raying the patient to confirm proper placement.	
**Flushing the catheter**			
	Syringe	Physicians faced challenges with maintaining proper syringe grip during aspiration and flushing. Physicians struggle with the current syringe design to alternate between 2 grips.	
	Valves	The T-shaped valves provided in the kit also present challenges when flushing the line after placement. The syringe often slips off the valve when flushing the lines.	
**Suturing and dressing placement**			
	Suture loop (feature) on the catheter	The suture loop anchor and box clamp were not always used. If there is excess catheter outside of the patient’s body, depending on the insertion location and the patient’s anatomy, the provider will use the suture loop anchor and box clamp.	

This contextual inquiry reveals that central venous catheterization is not merely a sequence of technically codified steps but also an intricate sociotechnical performance in which device design, environmental constraints, and clinician improvisation intersect to shape risk. Our mapping of 34 discrete actions, the dominance of preparation time in most settings, and the recurrent need to compensate for missing or poorly engineered components align with contemporary estimates that roughly 3% of patients exposed to a catheter for 3 days sustain a major complication [1]. Large contemporary reviews showcase that CVC is now routine, roughly 8% of hospitalized patients need a CVC, and more than 5 million are placed annually in the United States. Ultrasound-guided puncture should be the default. However, Kehagias et al [8] found that observations of obscured monitors, awkward one-handed aspiration, and improvised wire handling show how the safest technique on paper can still be undermined by poor ergonomics and kit design.

Our findings build on imperatives for field-based inquiry into real work conditions to surface latent design hazards. We observed exactly the kinds of use errors that are of concern: kinking guidewires without tactile feedback, dilators camouflaged against drapes, valves incompatible with existing hospital hardware, and ultrasound displays positioned outside the operator’s natural sightline. Such mismatches between device affordances and predictable human behavior may shift cognitive burden to clinicians, who must remember workarounds and coordinate ad hoc assistance while maintaining sterility. A contextual inquiry approach is designed to elicit these mismatches; our study demonstrates its value in an acute, invasive procedure where seconds and millimeters matter. The imperative to integrate these insights into formal design controls is clear.

Several concrete implications for device and kit redesign emerge from our contextual findings and accord with human factors guidance that interventions must fit users’ capabilities, workflows, and environments to sustain adoption and fidelity [9-12]. The suggested improvements and justifications are mentioned subsequently.

First, rather than perpetuating one-size-fits-all assortments, kits could be organized and sufficiently complete for the intended procedure and patient mix, thus minimizing secondary searches and redundant openings so that routinely needed sterile flushing media, local anesthetic, echogenic introducer needles of adequate length for patients classified as obese, and valve connectors compatible with local infusion hardware become immediately available; these specific component choices derive from our observations, whereas the general mandate to reduce cognitive and physical load is articulated in human-centered design literature [9,12].

Second, because the procedure advances in a sequential order, CVC kit components should be packaged in the same order to reduce cognitive burden. High-risk elements could incorporate salient visual and tactile affordances such as blood-tolerant depth markings on guidewires, textured feeders that signal advancement, and color-contrasted dilators that cannot visually disappear against drapes, an approach consistent with human factors recommendations to engineer cues that support rapid, accurate action under stress [12].

Third, ultrasound ergonomics require particular attention: practice reviews emphasize that the operator should keep the puncture site, needle, and image within a single line of sight, yet we repeatedly observed monitors positioned laterally or behind the user, encouraging awkward posture and one-handed aspiration [9-11].

Fourth, because maintaining stability of the introducer needle at the insertion site is critical, there is an opportunity for a device that minimizes hand movement or stabilizes the ultrasound probe during vessel access.

Fifth, converging evidence from simulation and ergonomic assessments shows that suboptimal screen and table positioning increases musculoskeletal strain and facilitates needle advancement errors, especially among novices, supporting the design of articulating mounts, probe‑holding accessories, and workstation layouts that free the dominant hand and keep the image within the operative field [10-12].

Sixth, additionally, as insertion components are introduced sequentially, combining the components associated with adjacent actions into a single integrated unit would significantly simplify the procedure.

Seventh, as threading the dilator over the guidewire is related to a risk of kinking, a device may reduce or eliminate the need for over-the-wire threading while reducing the risk of guidewire kinking.

Eighth, patient experience is also inseparable from considerations of safety. Drapes that blanket a conscious patient’s face created anxiety and impeded airway access, whereas Trendelenburg positioning with head rotation was difficult to sustain, particularly for individuals classified as obese, who then required additional supplies and time. Our observations suggest that rethinking drape architecture to permit facial exposure without compromising sterility and integrating oxygen delivery ports or transparent windows could mitigate claustrophobia and facilitate airway monitoring.

Because CLABSI remains among the costliest hospital-acquired infections on a per-case basis, the marginal gains from such design tweaks may translate into substantial economic and human benefit when scaled across millions of annual insertions [13]. Even in the era of prevention bundles, catheter dwell time remains a potent infection driver; CLABSI rates in one 2-year adult cohort climbed from 4.80 to 8.64 per 1000 catheter days as dwell exceeded 20 days, with multidrug resistant *Acinetobacter baumannii* predominating, mirroring some of our concerns that missing components, repeated kit openings, and ad hoc maintenance steps prolong setup and line life, thereby compounding exposure to contamination [14]. It has been previously emphasized that complication profiles can hinge on site choice, catheter caliber, and positioning—adult data (eg, 3SITES study) link subclavian access to fewer infections but more pneumothoraxes, whereas pediatric series show different risk patterns—and it has been reiterated that neutral rather than exaggerated positioning and meticulous ultrasound use can reduce failed passes and arterial hits, resonating with our field notes on stressful needle advancement angles, off-axis screens, and repeated punctures at the point deemed to be of the highest stress [15]. Other recommendations also somewhat align with our design proposals: select the smallest necessary lumen count; favor nontunneled catheters for fewer than 3 to 4 weeks and peripherally inserted central catheters when therapy exceeds 6 days; cap prolonged catheterization (approximately 14 days) to curb bacteremia; and mandate real-time ultrasound, chlorhexidine alcohol preparation, and daily site surveillance [16]. These were practices that our contextual inquiry somewhat related to and found were variably executed or actively hindered by kit incompleteness, unclear layer sequencing, and drapes that compromise both sterility and patient comfort. In a simulation of 40 anesthesia providers, patient safety (mean importance score 83.9/100), ease of use (mean score 64.6/100), and reduced clinician error (mean score 61.1/100) topped the selection criteria. A novel CVC system with a sequentially organized tray, enhanced labeling, and a guidewire funnel earned significantly higher scores for satisfaction overall, ease of use, layout, and safety (*P*≤.01 in all cases) and reduced 5 of 7 common risks (including clinician error and contamination or infection), aligning with the usability deficits (wire kinking, component hunting, and ambiguous tray hierarchy) that our study catalogued [7].

This study also highlights how waste and sharps handling were downstream consequences of kit design. The absence of designated receptacles for used guidewires and scalpels, the lack of sharps holders integrated into trays, and the routine opening of multiple kits for a single missing part create biohazard exposure and substantial nonrecyclable waste. While our qualitative approach did not quantify environmental impact, other health systems work has emphasized the financial and ethical importance of reducing unnecessary disposables; therefore, future prototypes should embed closed-loop sharps to capture and minimize redundant components to support infection prevention and sustainability goals simultaneously. This inference from our data warrants targeted life cycle and cost analyses in subsequent studies.

Viewed through the intersecting lenses of safety, efficacy, and usability for patients, providers, and products, our findings provide the basis for an argument that a device ecosystem should simultaneously consider the precepts mentioned subsequently.

The first precept is to shield patients from infection, vessel injury, and the cascade of “extra” procedures (intubation and deep sedation) by shortening setup and puncture time and by making correct J-wire orientation and tip control essentially foolproof.

The second precept is to protect clinicians from sharps and blood under tension, awkward postures, and protocol drift by embedding ergonomic grips; one-handed aspiration aids; and closed, labeled receptacles that keep contaminated instruments off ad hoc trays.

The third precept is to streamline the product itself with sequential, memory-light assembly; color and texture coding; and built-in safeguards (eg, wire funnels, depth markings visible through bloodied gloves, and lockable sharps wells) that are robust and attuned for real-world conditions such as low light; urgent timelines; moving patients; and gloved, fatigued hands. In effect, an intuitive kit that aligns with predictable human behavior and eliminates unnecessary multistep detours can reduce novice failure rates, cognitive load, and practice inconsistency, just as it could serve to curb nonrecyclable waste from “backup” kits opened for a single missing item.

By reframing CVC systems as sociotechnical solutions rather than static assortments of parts, the same design moves that minimize improvisation and coordination errors for physicians also reduce dwell time and manipulation events for patients, thereby advancing the core triad of safety, procedural efficacy, and everyday usability.

### Limitations

Our findings should be interpreted considering several limitations. This study was conducted in 3 US hospitals and included 19 observations and 24 interviews, which constrains generalizability, particularly to resource-limited settings or institutions that use different kit vendors. Although we captured real procedures, observer presence and video recording may have altered behavior, and some demonstrations on simulated patients cannot fully reproduce the stressors of an emergent cannulation. We did not measure clinical outcomes linked to the specific use problems we identified, nor did we quantify cognitive load or musculoskeletal strain. Furthermore, our sample comprised physicians; nurses, technicians, and infection preventionists often influence setup, maintenance, and postplacement care, and their perspectives warrant inclusion in a fuller systems analysis.

### Conclusions

Future work should transition from description to intervention. Rapid-cycle prototyping informed by these contextual insights, followed by high-fidelity simulation and usability testing in accordance with relevant guidelines, could generate validated design changes. Eye tracking, motion capture, and workload assessment tools may quantify how redesigned components alter gaze patterns, posture, and error rates. Multicenter trials comparing integrated, human-centered kits with current products could measure effects on insertion time, complication rates, waste generation, and cost. Furthermore, translating these methods to maintenance phases, including line access and dressing changes, could extend human factors benefits beyond placement to the complete catheter life cycle.

By examining how clinicians place central lines, this study reveals modifiable design flaws that perpetuate risk despite decades of procedural standardization. Contextual inquiry provides the evidentiary bridge between clinical imperatives to reduce complications and the practical realities of device use. Embedding such investigations at the outset of design and iteratively throughout product life cycles offers a path toward safer, more efficient, and more humane central venous access for both patients and providers.

## Abbreviations

CLABSI: central line–associated bloodstream infection
CVC: central venous catheter
ED: emergency department
ICU: intensive care unit
OR: operating room

## Data Availability

The datasets generated or analyzed during this study are not publicly available due to privacy and confidentiality purposes.
